# Assessment of Functional Recovery and Complications in Adult Diaphyseal Femur Fractures Treated With Closed Intramedullary Interlocking Nailing

**DOI:** 10.7759/cureus.85120

**Published:** 2025-05-31

**Authors:** Tariq Ahmad, Muhammad Tayyab, Muhammad Arsalan Azmat Swati, Kamran Khan, Rahim Khan, Khalid Khan

**Affiliations:** 1 Orthopedic Surgery, Medical Teaching Institution (MTI) Mardan Medical Complex, Mardan, PAK; 2 Trauma and Orthopedics, Bradford Teaching Hospitals NHS Foundation Trust, Bradford, GBR; 3 Orthopedics, Swat Paramedical Institute, Swat, PAK; 4 Orthopedics and Trauma, Medical Teaching Institution (MTI) Mardan Medical Complex, Mardan, PAK; 5 Orthopedics, Medical Teaching Institution (MTI) Mardan Medical Complex, Bacha Khan Medical College, Mardan, PAK

**Keywords:** femoral shaft fractures, functional outcome, harris hip score, interlocking nailing, orthopedic surgery, visual analog scale

## Abstract

Background: Adults often sustain high-energy injuries called diaphyseal fractures of the femur, which are typically treated surgically for the greatest cure.

Objective: This study evaluated the functional and radiological outcomes of adult diaphyseal femur fractures treated with closed intramedullary interlocking nailing over a 12-month follow-up using standardized clinical tools.

Methodology: This prospective observational study was conducted at the Department of Orthopedic Surgery, Bacha Khan Medical College, from January 2022 to December 2023. This study included 96 adult patients who had solitary diaphyseal femur fractures treated with closed intramedullary interlocking nailing. We gathered information on recovery parameters, fracture characteristics, and demographics. The Harris hip score and visual analog scale (VAS) were used to measure functional outcomes at six weeks, three months, six months, and one year. Data analysis was done using Statistical Package for the Social Sciences version 25.0 (IBM Corp., Armonk, NY).

Results: The average time to reach radiological union was 14.83 ± 3.12 weeks, and the time to full weight-bearing was 10.42 ± 2.65 weeks. The average range of motion was 122.3° ± 11.8° for the knee and 116.7° ± 10.5° for the hip. At one year, the mean Harris hip score increased from 71.45 ± 13.21 at six weeks to 85.27 ± 8.46, with 39.58% of patients having outstanding scores. The mean pain decreased from 5.27 ± 1.82 to 2.17 ± 1.35, indicating a substantial fall in VAS ratings as well. Sixteen patients (16.67%) had complications, with superficial infections accounting for most cases (n = 6; 6.25%). Transverse fractures (p = 0.035) and early weight-bearing (p = 0.038) were linked to better functional results.

Conclusion: Closed intramedullary interlocking nailing is an effective treatment for diaphyseal femur fractures, promoting early recovery with favorable functional outcomes and minimal complications.

## Introduction

Diaphyseal fractures of the femur are among the most serious and frequent long-bone injuries in adult trauma patients [1,2]. These fractures often result from high-energy trauma, such as road traffic accidents or falls from significant heights, and are typically associated with extensive soft tissue damage, considerable blood loss, and prolonged morbidity [3]. Given the femur's critical role in weight-bearing and ambulation, timely and effective treatment is essential to promote optimal healing and minimize long-term disability [4].

Over time, the surgical management of femoral shaft fractures has evolved considerably [5]. Closed intramedullary interlocking nailing has emerged as the gold standard due to its biomechanical stability, minimally invasive nature, high union rates, and facilitation of early mobilization [6]. This technique minimizes soft tissue disruption and maintains periosteal blood supply, thereby enhancing bone healing and reducing the risks of infection and nonunion [7,8].

Despite its widespread acceptance, functional outcomes following this procedure can vary significantly. Influencing factors include patient-related variables (age, comorbidities, and adherence to rehabilitation), fracture characteristics (location, pattern, and comminution), and surgical aspects (timing, technique, and choice of implant) [9,10]. While radiological union is a key milestone, comprehensive recovery encompasses pain relief, restoration of range of motion, return to work, and overall quality of life [11].

In resource-limited settings, disparities in rehabilitation and follow-up care further underscore the importance of evaluating not only the technical success of the procedure but also its real-world functional outcomes. Comprehensive assessments are essential to inform clinical practice, guide perioperative care, and support patient education initiatives.

Research objective

The study aimed to evaluate the functional and radiological outcomes of diaphyseal femur fractures in adult patients treated with closed intramedullary interlocking nailing using standardized clinical assessment tools (Harris hip score and visual analog scale, VAS) over a 12-month follow-up period. It also aimed to identify factors influencing postoperative recovery and complications.

## Materials and methods

Study design and setting

This prospective observational study was conducted in the Department of Orthopedic Surgery at Bacha Khan Medical College over a two-year period from January 2022 to December 2023. It involved adult patients who presented with radiologically confirmed solitary diaphyseal femur fractures and were treated with closed intramedullary interlocking nailing.

Inclusion and exclusion criteria

Inclusion criteria comprised adult patients (aged 18 years and above) with isolated, closed diaphyseal fractures of the femur treated with closed intramedullary interlocking nailing during the study period, who completed a minimum of 12 months of postoperative follow-up. Patients were excluded if they had open fractures, pathological fractures, polytrauma, or failed to complete the full follow-up period.

Sample selection

A total of 96 adult patients were enrolled in the study, all meeting the predefined inclusion criteria and completing the 12-month postoperative follow-up. Eligible patients were aged 18 years or older with isolated, radiologically confirmed diaphyseal femur fractures treated with closed intramedullary interlocking nailing. Inclusion was limited to individuals with complete clinical and radiological records across all designated follow-up intervals (six weeks, three months, six months, and one year). Patients were excluded if they had open or pathological fractures, associated polytrauma, prior femoral surgery or deformity, or if they were lost to follow-up or had incomplete documentation. All qualifying cases presenting during the study period were included consecutively, without random sampling.

Data collection

Demographic and clinical data, including age, gender, mechanism of injury, fracture classification, and laterality, were collected using a standardized proforma. Functional outcomes were assessed at six weeks, three months, six months, and 12 months postoperatively using the Harris hip score and VAS for pain. Additional outcome measures included time to radiological union, range of motion, time to full weight-bearing, and any postoperative complications such as infection, nonunion, or implant failure.

Statistical analysis

Data were analyzed using Statistical Package for the Social Sciences version 25.0 (IBM Corp., Armonk, NY). Descriptive statistics summarized patient characteristics and outcome measures, with continuous variables presented as means ± standard deviations and categorical variables as frequencies and percentages. Group comparisons involving two categories (e.g., age <40 vs. ≥40) were assessed using independent t-tests. For variables with more than two categories (e.g., fracture pattern: transverse, oblique, comminuted), one-way analysis of variance (ANOVA) with post hoc Tukey’s tests was performed to identify significant differences between the groups. Multiple linear regression analysis was conducted to identify independent predictors of the Harris hip score at one year to evaluate associations while adjusting for potential confounders. Predictor variables included age, gender, fracture pattern, mechanism of injury, time to full weight-bearing, and time to surgery. Model assumptions were checked and met prior to analysis, and only patients with complete data were included. A p value of less than 0.05 was considered statistically significant.

Ethical approval

Ethical clearance was obtained from the Institutional Review Board of Bacha Khan Medical College. Written informed consent was collected from all participants. Confidentiality and data anonymity were maintained throughout the study.

## Results

The general characteristics of the 96 patients included in the project are presented in Table 1. A significant portion of patients, 35.42% (n = 34 of 96), were between the ages of 18 and 30. Male patients constituted the majority of the study population, accounting for 72.92% (n = 70 of 96). The predominant cause of injury was road traffic accidents, which was reported in 66.67% (n = 64 of 96) of cases. The right femur was the more frequently injured side, occurring in 54.17% (n = 52 of 96) of patients. Regarding fracture patterns, transverse fractures were the most prevalent, observed in 37.50% (n = 36 of 96) of individuals. This was followed by comminuted fractures, which accounted for 32.29% (n = 31 of 96), and oblique fractures, present in 30.20% (n = 29 of 96) of patients. Statistical analysis showed significant associations using ANOVA between fracture pattern and Harris hip score (p = 0.035), and using independent t-tests for early weight-bearing and improved outcomes (p = 0.038), with significance set at p < 0.05.

**Table 1:** Demographic and clinical characteristics of the study population ^*^Statistically significant at p < 0.05 SD: standard deviation; ANOVA: analysis of variance

Variable	Categories	Number of patients, n (%)	Harris hip score, mean ± SD	Statistical test	p value
Age group (years)	18-30	34 (35.42%)	84.8 ± 7.2	ANOVA (post hoc Tukey)	0.041^*^
	31-45	29 (30.20%)	83.2 ± 8.1		
	46-60	19 (19.79%)	79.7 ± 9.2		
	>60	14 (14.59%)	78.1 ± 10.4		
Gender	Male	70 (72.92%)	82.7 ± 8.3	t-test	0.512
	Female	26 (27.08%)	81.5 ± 9.1		
Mechanism of injury	Road traffic accident	64 (66.67%)	83.9 ± 8.4	ANOVA	0.176
	Fall from height	21 (21.87%)	80.3 ± 9.0		
	Other	11 (11.46%)	81.1 ± 7.6		
Side of fracture	Right	52 (54.17%)	82.9 ± 8.1	t-test	0.668
	Left	44 (45.83%)	81.9 ± 9.0		
Fracture pattern	Transverse	36 (37.50%)	86.5 ± 7.2	ANOVA (post hoc Tukey)	0.035^*^
	Oblique	29 (30.20%)	82.0 ± 8.9		
	Comminuted	31 (32.29%)	78.6 ± 9.5		

The average time for patients to reach full weight-bearing was 10.42 ± 2.65 weeks, and radiological union was recorded at 14.83 ± 3.12 weeks (Table 2). A strong functional recovery in terms of joint mobility was shown by the mean range of motion, which was 122.3° ± 11.8° at the knee and 116.7° ± 10.5° at the hip.

**Table 2:** Postoperative recovery parameters SD: standard deviation

Parameter	Mean ± SD
Time to full weight-bearing (weeks)	10.42 ± 2.65
Time to radiological union (weeks)	14.83 ± 3.12
Range of motion at hip (degrees)	116.7 ± 10.5
Range of motion at knee (degrees)	122.3 ± 11.8

**Figure 1:**
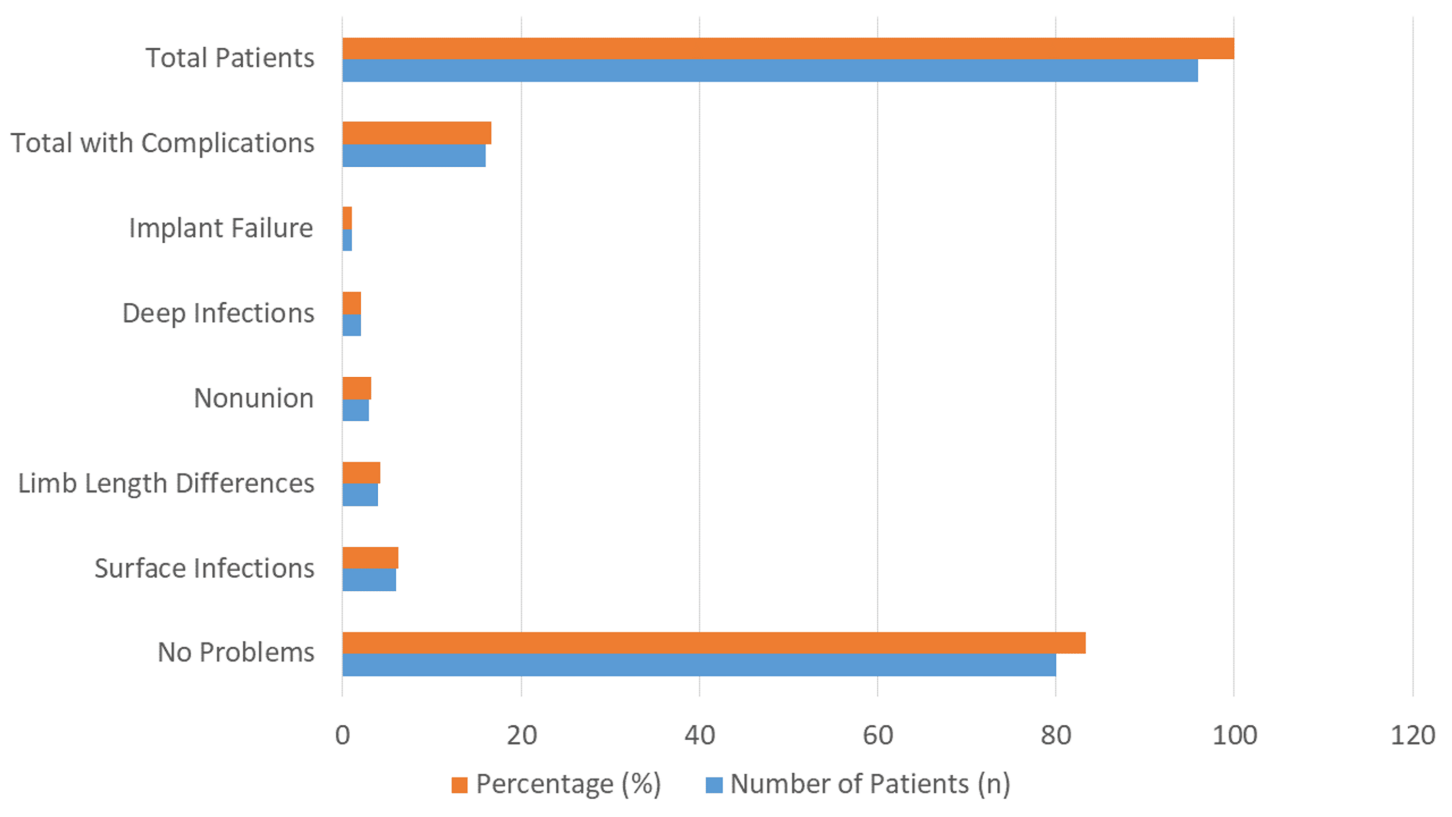
Complications observed during follow-up

Figure 1 illustrates the complications observed during the follow-up period. Notably, 80 out of the 96 patients (83.33%) experienced no complications. The overall rate of complications was low. Among the complications that did occur, surface infections were the most frequent, affecting 6.25% (n = 6 of 96) of the patients. Limb length discrepancies were reported in 4.17% (n = 4 of 96) of cases. Nonunion of the fracture occurred in 3.13% (n = 3 of 96) of patients, while deep infections were observed in 2.08% (n = 2 of 96). There was a single instance of implant failure, which corresponds to 1.04% (n = 1 of 96) of the study population.

As detailed in Table 3, Harris hip scores demonstrated a progressive improvement over the follow-up intervals. The proportion of patients categorized with poor scores (scores <70) significantly decreased from 29.17% (n = 28 of 96) at the six-week mark to 8.33% (n = 8 of 96) at the one-year follow-up. In contrast, the percentage of patients achieving excellent scores (scores 90-100) rose substantially, from 8.33% (n = 8 of 96) at six weeks to 39.58% (n = 38 of 96) at one year. The mean Harris hip score reflected this positive trend, increasing from 71.45 ± 13.21 at six weeks to 85.27 ± 8.46 at one year. Pain assessment, measured by the VAS, also showed considerable improvement. The mean VAS score decreased from 5.27 ± 1.82 at six weeks to 2.17 ± 1.35 at the one-year follow-up. Furthermore, the proportion of patients reporting no pain (VAS 0) increased from 12.50% (n = 12 of 96) at six weeks to 27.08% (n = 26 of 96) at one year.

**Table 3:** Functional outcomes and pain assessment at defined postoperative intervals VAS: visual analog scale

Outcome/time interval		Six weeks	Three months	Six months	One year
Harris hip score	Excellent (90-100)	8 (8.33%)	18 (18.75%)	30 (31.25%)	38 (39.58%)
	Good (80-89)	20 (20.83%)	30 (31.25%)	32 (33.33%)	32 (33.33%)
	Fair (70-79)	40 (41.67%)	28 (29.17%)	18 (18.75%)	18 (18.75%)
	Poor (<70)	28 (29.17%)	20 (20.83%)	16 (16.67%)	8 (8.33%)
	Mean Harris hip score	71.45 ± 13.21	78.12 ± 10.35	84.78 ± 7.26	85.27 ± 8.46
VAS	0 (no pain)	12 (12.50%)	18 (18.75%)	22 (22.92%)	26 (27.08%)
	1-3 (mild pain)	24 (25%)	36 (37.50%)	48 (50%)	48 (50%)
	4-6 (moderate pain)	32 (33.33%)	26 (27.08%)	16 (16.67%)	18 (18.75%)
	7-10 (severe pain)	28 (29.17%)	16 (16.67%)	10 (10.42%)	4 (4.17%)
	Mean VAS score	5.27 ± 1.82	4.02 ± 1.47	3.12 ± 1.08	2.17 ± 1.35

Table 4 demonstrates that there was no significant correlation between functional results and age, gender, or the type of injury (p > 0.05). However, early weight-bearing (≤10 weeks) was linked to improved outcomes (p = 0.038), and patients with transverse fractures had considerably better Harris hip scores than those with comminuted fractures (p = 0.035).

**Table 4:** Associations between functional outcomes and patient-related or surgical variables ^*^p value of <0.05 is significant SD: standard deviation

Variable		Harris hip score, mean ± SD	t-value	p value
Age group (years)	18-30	85.74 ± 7.01	1.42	0.164
	31-45	83.21 ± 6.23		
	46-60	78.55 ± 8.14		
	>60	71.29 ± 10.68		
Gender	Male	84.98 ± 7.35	1.52	0.131
	Female	81.45 ± 8.11		
Mechanism of injury	Road traffic accident	83.76 ± 7.84	1.38	0.174
	Fall from height	80.83 ± 6.42		
	Other	82.50 ± 7.89		
Fracture pattern	Transverse	86.21 ± 7.12	2.14	0.035^*^
	Oblique	83.21 ± 6.67		
	Comminuted	79.74 ± 8.52		
Time to full weight-bearing (weeks)	≤10	85.22 ± 6.51	2.12	0.038^*^
	>10	80.56 ± 7.99		

**Figure 2:**
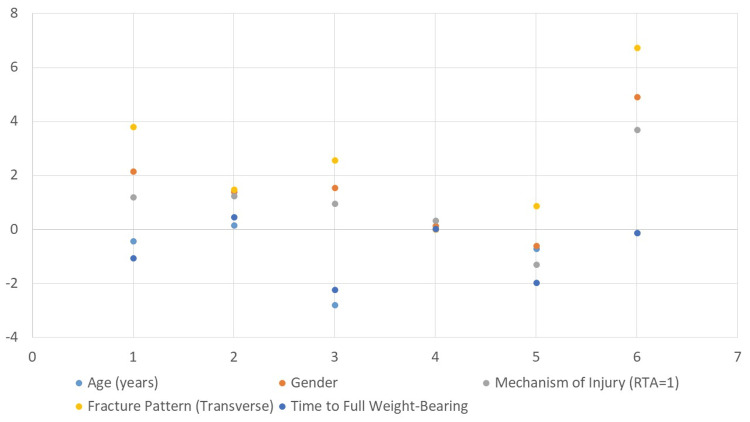
Multivariate linear regression analysis predicting Harris hip score Dependent variable: Harris hip score Significant predictors at p < 0.05

After adjusting for other factors in the model, increasing age was significantly associated with a decrease in Harris hip score (β = -0.42, p = 0.006), indicating poorer functional outcomes among older patients. The presence of a transverse fracture pattern independently predicted better Harris hip scores compared to other fracture types (β = 3.80, p = 0.012). Additionally, a longer time to achieve full weight-bearing was significantly linked with worse outcomes (β = -1.05, p = 0.028). Gender and mechanism of injury were not significant independent predictors in this multivariate model (Figure 2).

## Discussion

The current investigation evaluated the functional prognosis of diaphyseal femur fractures in adults managed with closed intramedullary interlocking nailing, demonstrating favorable healing patterns and functional recovery in the majority of cases. Radiological union occurred at a mean of 14.83 ± 3.12 weeks, while full weight-bearing was achieved in 10.42 ± 2.65 weeks. These findings are consistent with earlier literature, which reported union times ranging between 12 and 16 weeks following closed nailing procedures [12].

Sahu specifically noted radiological healing and early mobilization within 12-14 weeks, which, in most cases, are managed with interlocking nails [13]. The slight variation in union times across studies may be attributed to differences in patient demographics, fracture complexity, surgical expertise, and rehabilitation protocols. Additionally, early surgical intervention and structured physiotherapy likely contributed to faster mobilization in our cohort.

Joint mobility outcomes were promising, with mean hip and knee range of motion measuring 116.7° ± 10.5° and 122.3° ± 11.8°, respectively. These results align with prior findings where the average postoperative knee range exceeded 120° in uncomplicated recoveries [14]. Restoration of joint function is a critical marker of rehabilitation success, and our study reaffirms this, with most patients regaining adequate mobility within one year. Variability in range of motion across studies may stem from differing follow-up durations, adherence to physiotherapy, and surgical techniques (open vs. closed nailing).

Regarding postoperative complications, our study observed minimal adverse outcomes: 83.33% of patients experienced no complications. The most frequent issues included superficial infections (6.25%), limb length discrepancies (4.17%), and nonunion (3.13%). These values closely mirror findings in the literature, where superficial infections occur in 5%-7% of cases and nonunion in approximately 6% [15]. Cammas et al. [16] reported that complications were present in 26% of the cases.

Our observed implant failure rate of 1.04% also aligns with previous reports indicating implant-related issues in fewer than 2% of patients [17], including Deepak et al. [18], who reported that five out of 30 cases had superficial infection, four had shortening of the limb, and one had delayed union. The solitary incidence of implant failure in our study could be attributed to accurate surgical technique, stringent postoperative care, and timely follow-up, reaffirming the mechanical reliability of interlocking nailing systems in femoral shaft fractures.

Functional recovery, as measured by the Harris hip score, significantly improved from 71.45 ± 13.21 at six weeks to 85.27 ± 8.46 at one year. The proportion of patients attaining excellent outcomes (scores of 90-100) rose from 8.33% to 39.58% over the same period. These improvements are comparable to other studies that reported good to excellent hip function in a majority of patients treated with closed intramedullary nailing after one year [11,19].

Our subgroup analysis indicated that transverse fractures were associated with significantly better Harris hip scores (p = 0.035), while early weight-bearing (≤10 weeks) also correlated with improved outcomes (p = 0.038). These findings reinforce the conclusions of earlier investigations, emphasizing the biomechanical advantages of stable fracture patterns and the rehabilitative benefits of early mobilization in optimizing long-term functional recovery [18,20].

Study strengths and limitations

Closed intramedullary interlocking nailing demonstrates high efficacy and safety in treating adult diaphyseal femur fractures, with early mobilization and specific fracture patterns contributing to improved outcomes. A major strength of this study is its prospective design, use of standardized assessment tools (Harris hip score and VAS), and a consistent one-year follow-up, which together facilitated a robust evaluation of functional outcomes and complications. Although the study was conducted at a single tertiary care center, it is important to note that the hospital is a large, high-volume institution with over 30 practicing orthopedic surgeons. This diversity in surgical expertise helps mitigate the risk of individual surgeon bias and increases the generalizability of the findings within similar institutional settings.

All eligible patients who met the inclusion criteria were enrolled consecutively during the study period, which avoids the bias associated with convenience sampling and improves the representativeness of the sample. The relatively small sample size (n = 96) remains a limiting factor for subgroup analysis. The study excluded patients with open fractures and polytrauma, limiting the applicability of the findings to more complex trauma cases. Finally, the absence of a control group managed with alternative surgical techniques restricts the ability to directly compare treatment efficacy.

## Conclusions

Closed intramedullary interlocking nailing demonstrates high efficacy and safety in treating adult diaphyseal femur fractures, with early mobilization and specific fracture patterns contributing to improved outcomes. With most patients regaining full weight-bearing by 10.42 ± 2.65 weeks and achieving radiological union by 14.83 ± 3.12 weeks, the procedure yields excellent functional outcomes. Over the one-year follow-up, Harris hip scores progressively improved while VAS pain scores declined, indicating substantial recovery in mobility and pain relief. The low complication rate and positive associations with transverse fracture patterns and early weight-bearing further affirm the reliability and effectiveness of this surgical approach in standard orthopedic practice.
